# Does zinc oxide nanoparticles potentiate the regenerative effect of platelet-rich fibrin in healing of critical bone defect in rabbits?

**DOI:** 10.1186/s12917-022-03231-6

**Published:** 2022-04-02

**Authors:** Esraa Zalama, Gamal Karrouf, Awad Rizk, Basma Salama, Alaa Samy

**Affiliations:** 1 Department of Surgery, Anesthesiology and Radiology, Faculty of Veterinary Medicine, Mansoura University, Mansoura, 35516 Egypt; 2 Department of Biochemistry and Chemistry of Nutrition, Faculty of Veterinary Medicine, Mansoura University, Mansoura, 35516 Egypt

**Keywords:** Zinc oxide, Nanoparticles, Platelet-rich fibrin, Critical bone defect, Canalization, Bridging, Remodeling, Bone density

## Abstract

**Background:**

Many encouraging studies confirmed the ability of Zinc Oxide Nanoparticles (ZnONPs) in accelerating bone growth and mineralization. The use of Platelet Rich-Fibrin (PRF) as a sole filling material for large segmental bone defects remains questionable. The objectives are to investigate the regenerative efficacy of autologous Platelet Rich-Fibrin (PRF) and Zinc Oxide Nanoparticles (ZnONPs) in repairing large segmental bone ulnar defects in a randomized controlled study in rabbits using computed tomographic interpretations. A 12 mm critical size defect was surgically induced in the ulna of 30 rabbits (*n* = 10/ group). In the control group, the defect was left empty. In the PRF group, the defect is filled with PRF. In the PRF/ZnONPs group, the defect is filled with PRF that was inoculated with 0.1 ml of 0.2% ZnONPs. Radiologic healing capacity was evaluated at the first, second, and third postoperative months.

**Results:**

Statistical analysis showed significant differences in the radiologic healing scores between the groups (*P* = 0.000–0.0001) at all-time points (*P* = 0.000–0.047) during the study.

**Conclusion:**

Rabbits in the PRF/ZnONPs group showed the highest appreciable bone quality and quantity followed by the PRF group with high quantity but low bone quality meanwhile, rabbits in the control group showed minimal quantity but medium bone quality. Interestingly, the addition of ZnONPs to PRF can accelerate the healing of ulnar critical-size defects in rabbits.

## Study design

A 12 mm critical size defect was created in the diaphysis of the right ulnae in thirty healthy male white New Zealand rabbits (*n* = 10/ group). In the control group, the defect was left for healing without grafts. In the platelet rich-fibrin (PRF) group, the defect is filled with autologous PRF clot. In the combination (PRF/ZnONPs) group, the defect is filled with autologous PRF clot inoculated with 0.1 ml of 0.2% ZnONPs dispersion. Radiologic healing capacity between the groups was evaluated by immediate postoperative (PO) radiologic assessment and subsequently at the first, second, and third postoperative months.

## Background

Critical size bone defect (CSD) is defined as the smallest intraosseous wound that does not heal spontaneously and there would never be complete bony regeneration despite surgical stabilization [1]. Despite it depends on the bone and the species of animal, the minimal size that renders the defect to be critical could be substantially determined as a segmental bone deficiency of a length exceeding 2–2.5 times the diameter of the affected bone and usually requires surgical intervention and bone reconstruction [2]. In veterinary practice, bone losses due to severe orthopedic trauma, oncologic resection, extensive debridement, and congenital malformations mostly lead to disability and dysfunction [3, 4].

The limitation of available reconstruction methods obligates resorting to tissue engineering and regenerative medicine to achieve the goal of bone augmentation and complete bone regeneration that cannot occur in the absence of osteogenic or osteoinductive bone materials [5, 6]. Several studies have shown that bone regenerative procedures may be accelerated by the addition of biomaterial containing specific growth factors. These growth factors play a central role in hemostasis, fibroblast mitogenesis, angiogenesis, macrophage activation, and cell proliferation, and subsequently, the bone healing process by inducing neovascularization and chemotaxis, and stimulating collagen synthesis [7, 8].

Platelet**-**rich fibrin (PRF) is an autologous strong fibrin clot rich in platelets, fibrin; leukocytes; circulating stem cells; healing cytokines; the pro-inflammatory cytokines interleukin-1ß, interleukin-6, and tumor necrosis factor-α; the anti-inflammatory cytokines interleukin-4 and interleukin-10; vascular endothelial growth factor; insulin-like growth factor-I and II; epidermal growth factor, transforming growth factor-β1 and platelet-derived growth factor [9]. Additionally, PRF allows significant postoperative protection of the surgical site and seems to accelerate the integration, maturation, and remodeling, while enhancing bone graft density if combined with it [10].

Zinc oxide nanoparticles (ZnONPs) are low toxic inorganic zinc-containing metal oxide nanoparticles that can be easily obtained through various methods and have received great interest in many fields, such as pollution treatment, food packaging, and biomedicine because of their ultra-violet filter properties and photochemical, antifungal, high catalyst and anticancer activities [11]. ZnONPs were used as excellent substitutes for antibiotics against multiple drug-resistant microorganisms and also possess osteogenic properties as they can accelerate bone growth and mineralization [12, 13].

Cone-beam computed tomography (CBCT) is a relatively recent CT modality that uses a cone X-ray beam instead of the conventional fan-beam and is widely used for the clinical diagnosis of dental complications for its advantages over the conventional CT as relatively lower cost and radiation dose but the higher spatial resolution [14].

Although PRF has been used in several critical bone defects, to the best of our knowledge, no study has reported the use of PRF as a sole filling biomaterial or even with ZnONPs for long bone critical segmental bone loss. Therefore, this study aimed to evaluate the osteogenic efficacy of autologous PRF alone or in combination with ZnONPs for the repair of induced segmental critical-sized ulnar bone defects in a rabbit model using cone-beam computed tomographic interpretations.

## Results

**Fig. 1:**
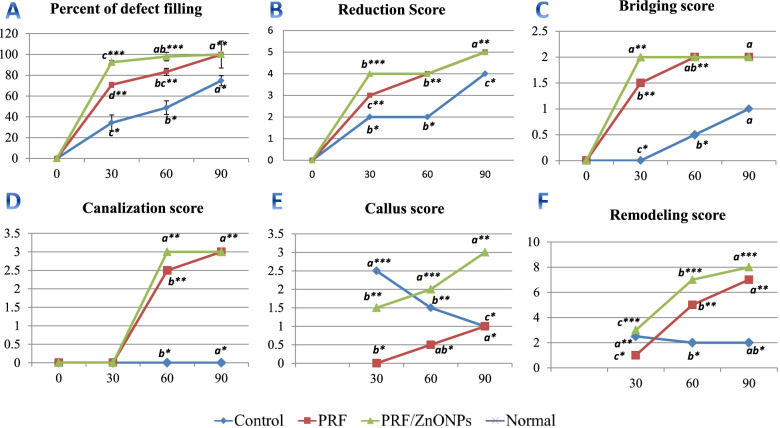
Graphs showing the Semi-quantitative (% of defect filling) and qualitative (scores) evaluation of newly bone formation using CBCT at the 30^th^, 60^th^ and 90^th^ days

All rabbits regained independent feeding ability with a normal gait, following recovery from anesthesia. No apparent signs of local infection were observed, other than two rabbits in the PRF and PRF/ZnONPs groups which suffered from infection and were excluded and replaced. The addition of biological grafts to a 12 mm segmental ulnar CSD showed remarkable radiological changes (Fig. 1 and Table 1), where statistical analysis showed significant differences in all CBCT evaluation criteria among the three experimental groups (*P* = 0.000–0.001) at all time-points (*P* = 0.000–0.047) during the study.

**Table 1:** Semi-quantitative (% of defect filling) and qualitative (scores) evaluation of newly bone formation using CBCT at the 30th, 60th and 90th days

Groups	30th	60th	90th	Kruskal-Wallis H	***P***-value
**Percent of defect filling (%)**					
Control	34.25 ± 7.58 ^c*^	48.73 ± 4.87^b*^	74.75 ± 4.69^a*^	–	0.000
PRF	70.87 ± 2.30^d**^	83.23 ± 12.98^bc**^	99.83 ± 0.41^a**^	–	0.000
PRF/ZnONPs	92.50 ± 1.58^c***^	97.9 ± 1.3 ^ab***^	100.0 ± 0.0^a**^	–	0.000
P-value	0.000	0.000	0.000		
**Reduction score**					
Control	2.0(2.0–2.0)^b*^	2.0(2.0–3.0)^b*^	4.0(3.0–4.0)^a*^	13.96	0.001
PRF	3.0(3.0–3.0)^c**^	4.0(3.0–4.0)^b**^	5.0(4.0–5.0)^a**^	14.61	0.001
PRF/ZnONPs	4.0(4.0–4.0)^b***^	4.0(4.0–5.0)^b**^	5.0(5.0–5.0)^a**^	13.69	0.001
Kruskal-Wallis H	17.00	13.69	13.65		
P-value	0.000	0.001	0.001		
**Bridging score**					
Control	0.0(0.0–0.0)^c*^	0.5(0.0–1.0)^b*^	1.0(1.0–1.0)^**a**^	15.33	0.000
PRF	1.5(1.0–2.0)^b**^	2.0(1.0–2.0)^ab**^	2.0(2.0–2.0)^**a**^	6.30	0.043
PRF/ZnONPs	2.0(1.0–2.0)^a**^	2.0(2.0–2.0)^a**^	2.0(2.0–2.0)^**a**^	4.18	0.124
Kruskal-Wallis H	17.89	19.56	23.00		
*P*-value	0.000	0.000	0.000		
**Canalization score**					
Control	0.0(0.0–0.0)^**b**^	0.0(0.0–0.0)^b*^	0.0(0.0–1.0)^a*^	6.57	0.037
PRF	0.0(0.0–0.0)^**c**^	2.5(2.0–3.0)^b**^	3.0(3.0–3.0)^a**^	19.93	0.000
PRF/ZnONPs	0.0(0.0–0.0)^**b**^	3.0(2.0–3.0)^a**^	3.0(3.0–3.0)^a**^	21.44	0.000
Kruskal-Wallis H	0.000	18.72	22.14		
P-value	1.000	0.000	0.000		
**Callus score**					
Control	2.5(2.0–3.0)^a***^	1.5(1.0–2.0)^b**^	1.0(0.0–1.0)^c*^	16.51	0.000
PRF	0.0(0.0–1.0)^b*^	0.5(0.0–1.0)^ab*^	1.0(0.0–1.0)^a*^	6.11	0.047
PRF/ZnONPs	1.5(1.0–2.0)^b**^	2.0(2.0–3.0)^a***^	3.0(2.0–3.0)^a**^	12.39	0.002
Kruskal-Wallis H	18.81	16.73	18.73		
*P*-value	0.000	0.000	0.000		
**Remodeling score**					
Control	2.5(2.0–3.0)^a**^	2.0(2.0–2.0)^b*^	2.0(2.0–3.0)^ab*^	6.30	0.043
PRF	1.0(0.0–2.0)^c*^	5.0(3.0–6.0)^b**^	6.0(5.0–6.0)^a**^	18.23	0.000
PRF/ZnONPs	3.0(3.0–4.0)^c***^	7.0(7.0–8.0)^b***^	8.0(7.0–8.0)^a***^	18.26	0.000
Kruskal-Wallis H	18.20	21.75	21.89		
*P*-value	0.000	0.000	0.000		

^a,b,c^Medians and ranges with different small superscripts letters in the same row are significantly different at *P* < 0.05

^*,**,***^Medians and ranges with different asterisks superscripts in the same column are significantly different at *P* < 0.05

**Fig. 2:**
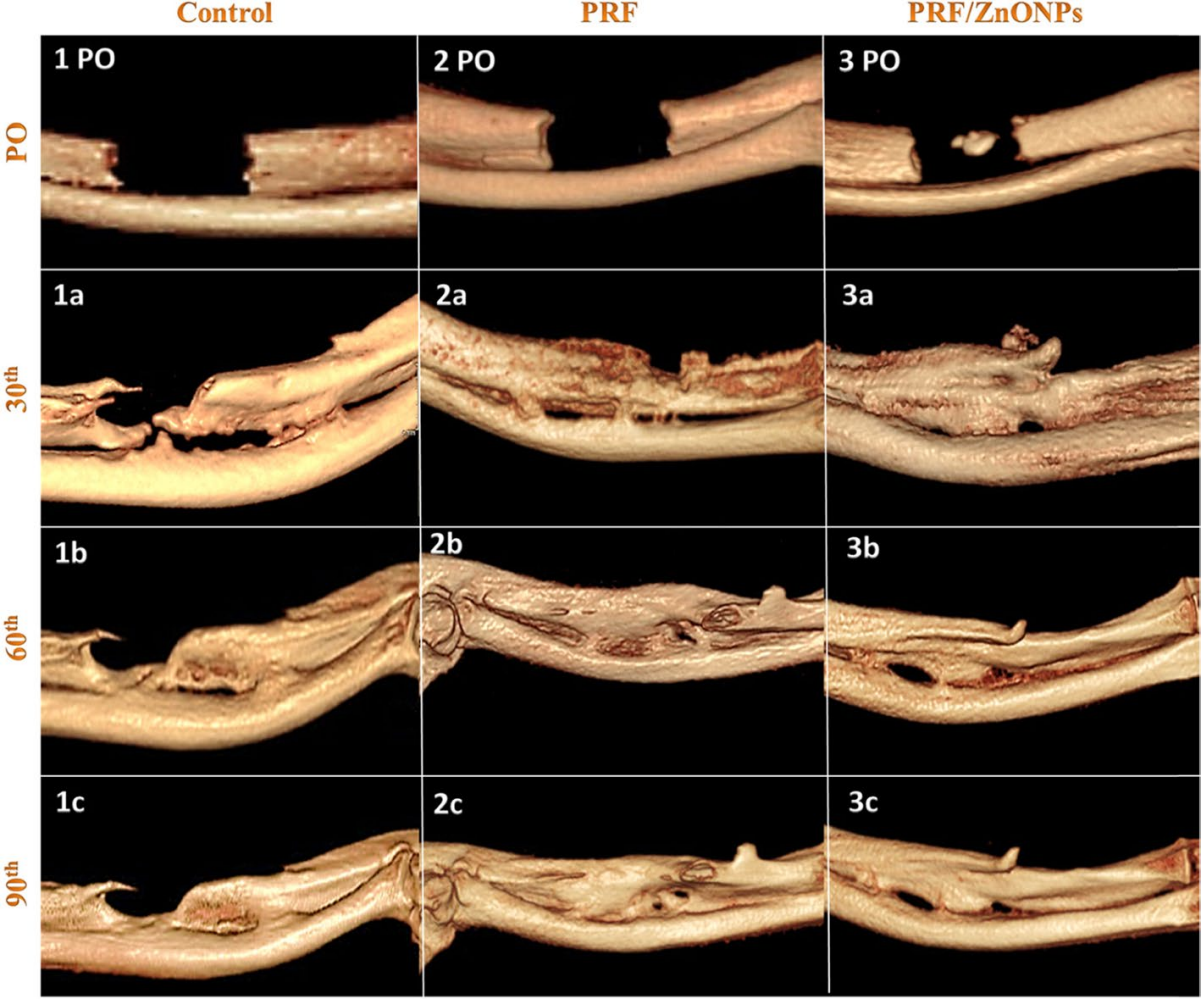
Computed tomography 3D images of the ulna with CSD in control (1PO-1c), PRF (2PO-2c) and PRF/ZnONPs (3PO-3c) at the 1^st^ (PO), 30^th^ (**a**), 60^th^ (**b**) and 90^th^ (**c**) postoperative days showed a highly significant increase in the percent of CSD filling rate in both PRF and PRF/ZnONPs groups versus the control group

Concerning the quantitative evaluation of new bone formation in the CSD, treatment either by PRF either alone (PRF group) or in combination with ZnONPs (PRF/ZnONPs group) revealed a highly significant (*P* = 0.000) increase in the percent of CSD filling rate versus the control group (Fig. 2). On the 30th PO day, the percent of defect filling was 70.87 ± 2.30% and 92.50 ± 1.58%, respectively versus 34.25 ± 7.58% in the control group, while at the 60th PO day; the percent of defect filling was 83.23 ± 12.98% and 97.87 ± 1.25% respectively, versus 48.73 ± 4.87% in the control group. At the 90th PO day, complete filling of the CSD was non-significantly observed in both treatment groups where the filling percent was 99.83 ± 0.41% and 100.0 ± 0.0%, respectively, versus 74.75 ± 4.69% in the control group. Accordingly, the reduction score of CSD was significantly (*P* = 0.000) high in PRF and PRF/ZnONPs groups versus the control group. The maximal reduction in the defect size was firstly recorded in the PRF/ZnONPs group on the 60th PO day.

**Fig. 3:**
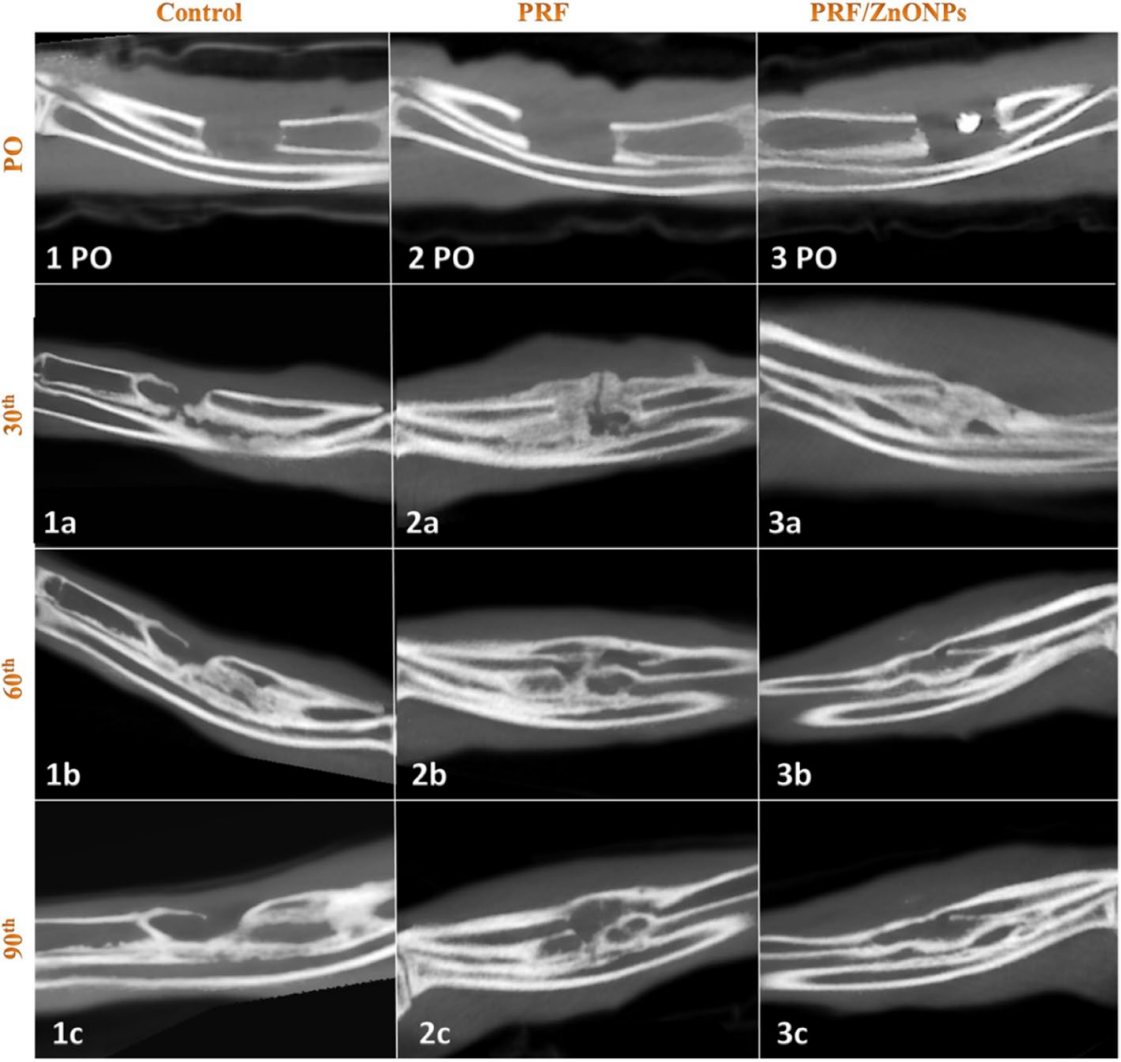
Computed tomography sagittal plane images of the ulna with CSD in control (1PO-1c), PRF (2PO-2c) and PRF/ZnONPs (3PO-3c) at the 1^st^, 30^th^ (**a**), 60^th^ (**b**) and 90^th^ (**c**) postoperative days showed complete bicortical bridging of the CSD in both PRF and PRF/ZnONPs groups versus unicortical bridging in the control group. Notice abundant callus formation in PRF group versus both control and PRF/ZnONPs groups

**Fig. 4:**
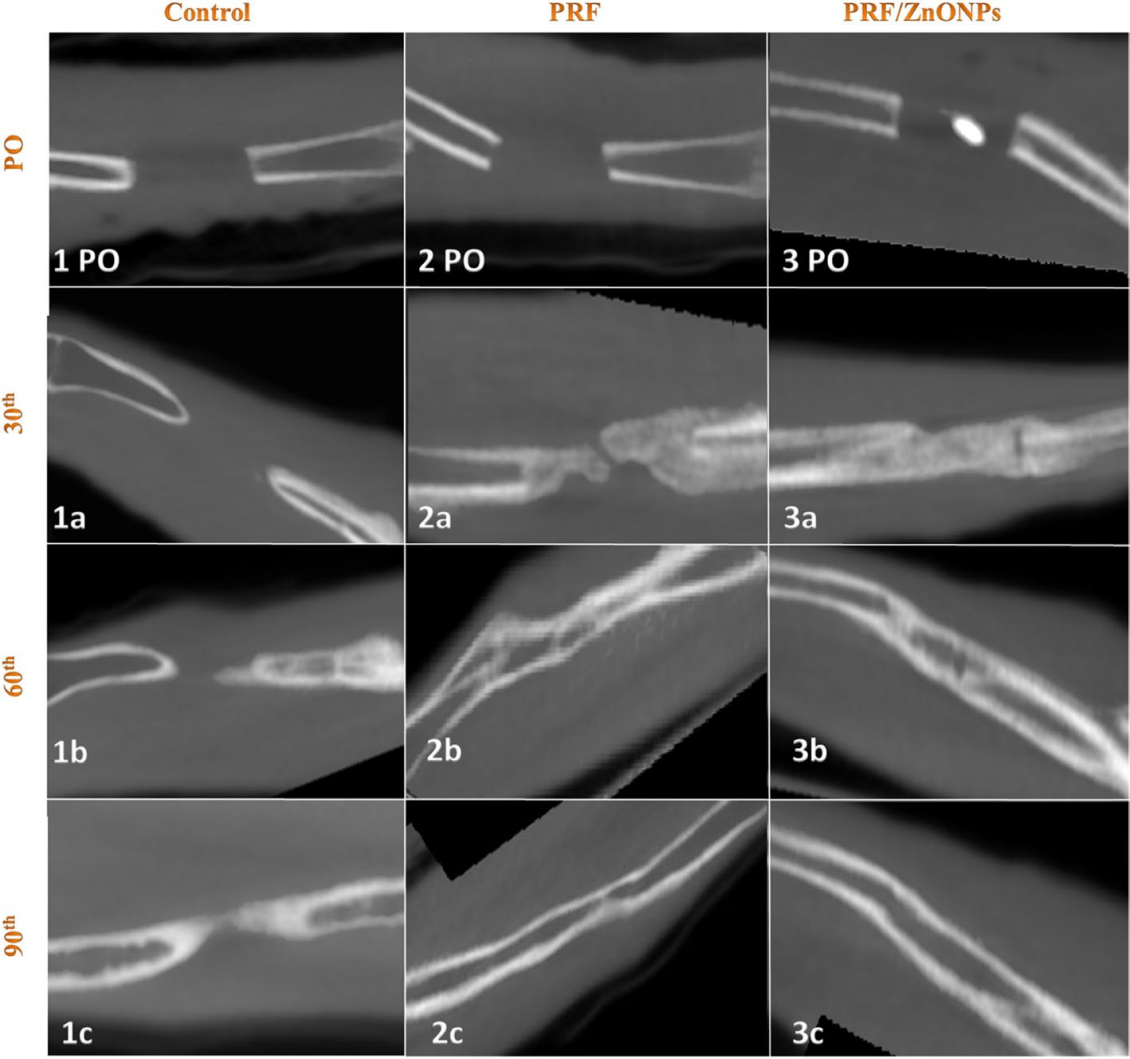
Computed tomography axial plane images of the ulna with CSD in control (1PO-1c), PRF (2PO-2c) and PRF/ZnONPs (3PO-3c) at the 1^st^, 30^th^ (**a**), 60^th^ (**b**) and 90^th^ (**c**) postoperative days showed recreation of the bone marrow cavity in both PRF and PRF/ZnONPs groups versus absence of canalization in the control group

**Fig. 5:**
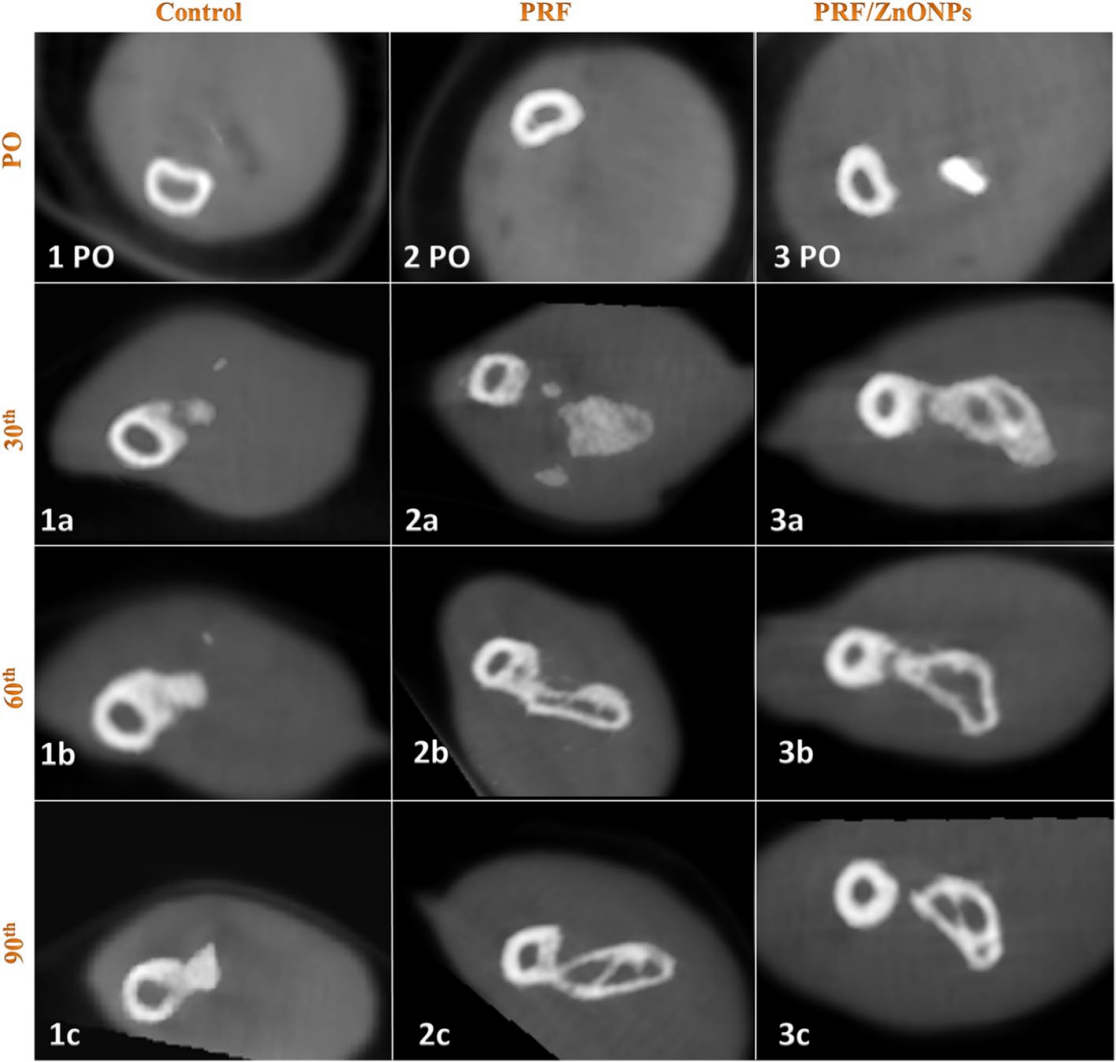
Computed tomography cross section images of the ulna with CSD in control (1PO-1c), PRF (2PO-2c) and PRF/ZnONPs (3PO-3c) at the 1^st^, 30^th^ (**a**), 60^th^ (**b**) and 90^th^ (**c**) postoperative days showed extensive fusion between the ulna and radius with incomplete remodeling of the defect site in both PRF and control groups versus regression of the bone in the interosseous space between radius and ulna and a reduction in the callus size in the PRF/ZnONPs group indicating better remodeling

In terms of the bridging score (Fig. 3), complete bicortical bridging of the CSD was observed in both treatment groups as early as the 30th PO day versus no bridging in the control group. All rabbits (*n* = 10/10) obtained complete bridging at the 60th and 90th PO days in both PRF/ZnONPs and PRF groups, respectively. Unicortical bridging was obtained in the control group from the 60th day until the end of the study. Regarding the canalization score (Fig. 4), the recreation of the bone marrow cavity wasn’t started at all in the control group meanwhile, it started after the 30th PO day in both the treatment groups with significant differences between groups (*P* = 0.000) within all time-points (*P* = 0.000–0.037). A high canalization score was recorded in both treatment groups on the 60th PO day and a maximal canalization score was obtained on the 90th day in both treatment groups. Referring to the callus score (Fig. 3), rabbits in PRF/ZnONPs group expressed the minimal callus size while rabbits in the PRF group showed the maximal callus size. On the 90th PO day, statistical analysis showed a non-significant difference in callus score between both control and PRF groups. Results of this study showed that both time and treatment had a significant impact (*P* = 0.000–0.043) on ulna remodeling after the experimental establishment of CSD. High appreciable remodeling scores (scores = 3, 7, and 8) were observed in the animals of the PRF/ZnONPs group on the examined time points respectively, versus both in the PRF group (scores = 1, 5, and 6) and the control group (scores = 2.5, 2, and 2). The coronal CBCT images (Fig. 5) showed regression of the bone in the interosseous space between radius and ulna and a reduction in the callus size in the PRF/ZnONPs group indicating better remodeling versus both PRF and control groups.

**Fig. 6:**
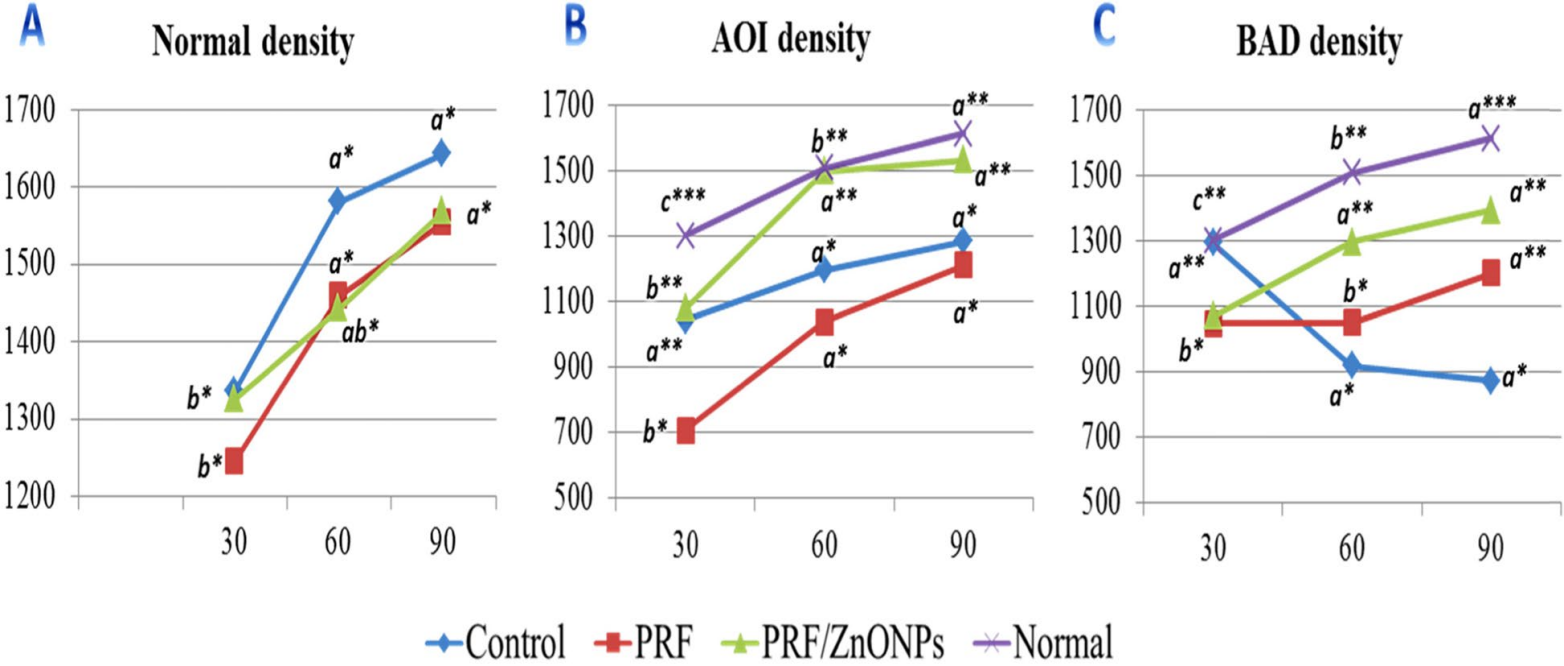
Graphs showing quantitative bone densities of normal bone, new bone in-growth density (AOI density), and density of bone adjacent to the defect area (BAD density) in HU obtained by CBCT and expressed as mean ± SD at the 30^th^, 60^th^ and 90^th^ days

Concerning the quantitative measurement of the bone densities (Fig. 6 and Table 2), results showed that time had a direct proportion significant effect on increasing the densities of normal ulnar bone (*P* = 0.000–0.012). Referring to the treatment method, results showed that the AOI of the PRF group had the relatively least bone density among the three groups at all time-points, while AOI of the PRF/ZnONPs group had the significant maximal (*P* = 0.000–0.009) density among the groups at all time-points. Consequently, when comparing the AOI densities of each group at each time to the normal ulnar densities, results showed that PRF/ZnONPs group displayed non-significant differences from the normal ulnar bone density on both the 60th and 90th PO day. Time showed a direct proportional significant effect on increasing the AOI’s bone densities in both the PRF and the PRF/ZnONPs groups (*P* = 0.000), versus the control group (*P* = 0.186). Both the control and the PRF groups were non-significant from each other in terms of AOI density from the 60th PO day till the end of the study.

**Table 2:** Quantitative bone densities of normal bone, new bone in-growth density (AOI density), and density of bone adjacent to the defect area (BAD density) in Houns Field units (HU) obtained by CBCT and expressed as mean ± SD at the 30th, 60th and 90th days

Groups	30th	60th	90th	***P*** value
**Bone densities (Houns Field units)**				
**Normal density**				
Control	(1335.75 ± 67.20)^b*^	(1582.03 ± 79.97)^a*^	(1643.75 ± 20.45)^a*^	0.000
PRF	(1245.83 ± 125.49)^b*^	(1460.43 ± 177.28)^a*^	(1554.73 ± 250.56)^a*^	0.012
PRF/ZnONPs	(1326.04 ± 134.37)^b*^	(1443.56 ± 157.81)^ab*^	(1569.52 ± 213.89)^a*^	0.004
*P* value	0.241	0.127	0.613	
Total Mean	(1302.54 ± 115.58)^**c**^	(1508.20 ± 144.52)^**b**^	(1612.69 ± 172.18)^**a**^	0.000
**AOI density**				
**Normal density**	(1302.54 ± 115.58)^c***^	(1508.20 ± 144.52)^b**^	(1612.69 ± 172.18)^a**^	0.000
Control	(1042.32 ± 181.63)^a**^	(1197.86 ± 218.81)^a*^	(1284.53 ± 188.30)^a*^	0.186
PRF	(706.45 ± 136.31)^b*^	(1037.48 ± 147.88)^a*^	(1212.52 ± 79.18)^a*^	0.000
PRF/ZnONPs	(1079.10 ± 48.41)^b**^	(1498.95 ± 77.19)^a**^	(1534.34 ± 111.98)^a**^	0.000
*P* value	0.000	0.000	0.009	
**BAD density**				
**Normal density**	(1302.54 ± 115.58)^c**^	(1508.20 ± 144.52)^b**^	(1612.69 ± 172.18)^a***^	0.000
Control	(1295.00 ± 15.56)^a**^	(919.70 ± 243.53)^a*^	(873.86 ± 150.03)^a*^	0.104
PRF	(1049.20 ± 59.48)^b*^	(1050.00 ± 49.49)^b*^	(1201.00 ± 15.56)^a**^	0.027
PRF/ZnONPs	(1070.80 ± 00.00)^b*^	(1297.50 ± 3.53)^a**^	(1394.00 ± 66.47)^a**^	0.008
*P* value	0.000	0.000	0.000	

^a,b,c^Medians and ranges with different small superscripts letters in the same row are significantly different at P < 0.05

^*,**,***^Medians and ranges with different asterisks superscripts in the same column are significantly different at *P* < 0.05

The density of the bone adjacent to the defect (BAD) was significantly different between all groups (*P* = 0.000). Passage of time was shown to have a significant impact (*P* = 0.008–0.027) on the BAD density in both PRF and PRF/ZnONPs groups versus the control group (*P* = 0.104). Both treatment groups were the most negatively affected groups on the 30th day while BAD density in the control group was the least affected and the nearest to the normal density. On the 60th day, the BAD density of the PRF/ZnONPs group had improved to be non-significant from the normal density while both the control and PRF groups were the most affected and non-significant from each other. On the 90th postoperative day, the BAD density of the control group was the least among the groups while it was non-significantly different between both PRF and PRF/ZnONPs groups. As an overall view, all BAD densities in all experimental groups were less than the mean of normal bone density at all time-points but an ascending manner was observed in both treatment groups along with the study while a descending manner was observed in the control group. A significant moderate negative correlation (spearman’s rho = − 0.547, *P* = 0.006) was observed between the BAD density and the level of callus score. In the control group, the callus increased significantly by the time and the BAD density decreased by the time. Meanwhile, in both PRF and PRF/ZnONPs groups the callus score decreased with time and the BAD density increased.

## Discussion

Bone repair in refractory cases of bone losses had represented a major difficulty in both veterinary and human practice. The presence of a large gap interrupts the progression of bone healing unless filling the gap with bone grafts or using distraction osteogenesis. The standard previous treatment modalities exhibit extremely high complication rates [2, 15]. Many efforts to develop new treatment modalities for segmental bone loss have resulted in designing new biodegradable implants that can incorporate novel osteogenic, osteoinductive, and/ or osteoconductive bone healing augmentation materials.

Ulna was selected in the present study because the fibro-osseous union with the adjoining radius proximal and distal to the surgical site is strong enough to act as a natural internal splint and support the animal during weight-bearing [16, 17]. Additionally, the relatively low cost, due to neither external nor internal fixation for the non-weight bearing ostectomized ulna, motivates this selection. Because the defect size is one of the substantial factors determining the degree and rate of bone union besides animals’ age, weight, and gender [18], a CSD of 12 mm diameter was selected after Gugala et al. [2] who declared that the segmental bone deficiency of a length exceeding 2–2.5 times of the diameter of the long bone can be considered the minimal size that renders the defect to be critical and usually requires surgical intervention and bone reconstruction. Generally, a bone osteotomy can be performed either by drilling [19, 20] or sawing [21, 22]. In this study, low-speed drilling was used under constant irrigation with normal saline to prevent thermal necrosis of the defect edges. After removal of the ulna segment, flushing of the defect with saline was mandatory in all groups to remove any resultant drilled bone swath materials which were used by many authors [23, 24] as morselized bone chips for their osteoinductive properties. Thus they could falsely affect the results and disturb radiographs and CT images.

From different bone tissue engineering models, PRF had been selected after several encouraging clinical studies which utilized PRF alone in CSD [23] or with bone cement on segmental bone defects [25], and resulted in better bone regeneration and good restoration of osteoblast attachment and subsequently bone healing [26, 27]. Additionally, autologous PRF that is obtained from the patient’s own blood contains several growth factors that contribute to tissue regeneration and healing in the defect area [28]. Furthermore, there is rare immunologic rejection and complications [29]. Most studies utilized the sole PRF for soft tissue repair [30] while using it as the sole filling material for large segmental bone defects remains questionable. Natural polymers such as gelatin, chitosan, fibrin, and collagen are commonly used along with each other or even as sole scaffolding materials in bone regenerative medicine [31, 32]. Consequently, we assumed that PRF can be used as a sole bio-scaffold for filling the ulnar segmental CSD with no need for additional solid osteoconductive material since fibrin matrix is the main constituent of PRF and ulna is a non-weight bearing bone. In this study, PRF was used for the first time in the treatment of segmental CSD without bone cement or bone graft. In our study, 4 ml of whole blood was used after Akyildiz et al. [33] for the preparation of autologous PRF for each animal and the eventual PRF clot was appropriate to the ulnar defect size as it wasn’t too small to prevent PRF-bone contact or too large to hinder the closing of the surrounding muscles without being ruptured.

Nanoparticles had been chosen to be involved in this study from the point of their rapidly growing concepts in tissue engineering for bone regeneration as mentioned by Mirzaei and Darroudi [34]. ZnONPs were selected because of their low toxicity, low cost and its availability compared to the other different natural non-organic metallic NPs [35], and Zn^2+^ ions are well-known to stimulate bone formation and mineralization and to have an active role in the proliferation of osteoblastic cells [36]. Although the low toxicity of ZnONPs, their solubility may contribute to cytotoxicity, oxidative stress, and mitochondrial dysfunction [37]. Therefore, their dispersion was diluted to 0.2% as recommended by Fielding et al. [38].

The three major properties that influence the selection of bone graft and its ability to promote fusion are osseointegration, osteoinduction, and osteoconduction and they must be completely biocompatible to reveal satisfactory results [39, 40]. Our experimental results showed that PRF and PRF/ZnONPs have osteogenic potential expressed by early reduction of the defect size, bicortical bridging of the defect, and recreation of the marrow cavity. Osseointegration was defined as the direct contact between living bone and implant at the light microscope level [41]. The osseointegration property of PRF could be attributed to the high tensile strength of the PRF clot in addition to its adhesiveness and stability [42]. Osteoinduction is the ability to produce signals that encourage primitive, undifferentiated, and pluripotent progenitor cells to differentiate into active osteoblasts. Zhou et al. [23] mentioned that PRF is considered a good medium for proliferation, differentiation, migration, and mineralization of cells during bone formation due to the progressive release of growth factors for more than 14 days in conjunction with fibrin net degradation after PRF application [43]. In terms of osteoconductivity, the ability to provide a scaffold for bone growth and vascularization [41], PRF can be considered as an excellent osteoconductive medium due to the fibrin matrix that exhibited an optimal environment for mesenchymal stem cells to adhere and differentiate [44–46]. From the same concept, great attention was paid to ensure graft**-**bone contact in order to obliterate any space which could prevent the osteoconductive property of the PRF.

All results of this study showed the superiority of the PRF/ZnONPs combination over the sole PRF clot in stimulation of bone healing in segmental CSD expressed by a faster reduction in defect size, higher bone density, lower callus size, and better remodeling with earlier canalization. Zhang et al. and Toledano et al. [12, 47] confirmed the ability of ZnONPs to enhance the osteogenic differentiation of the stem cells thus accelerating bone growth and mineralization. Zinc is an essential trace element naturally located at sites of tissue calcification, including osteons and calcified cartilage, and as bone mineralization increases, levels of zinc in bone tissue increase [48]. The alkaline phosphatase enzyme uses zinc as a cofactor involved in bone mineralization [49]. Zn^2+^ can also promote bone formation, growth, and mineralization [50]. All nanoparticles including ZnONPs can penetrate smaller blood capillaries and are amenable to be absorbed by the cells, allowing an efficient drug delivery to the target sites [51]. The biodegradable ZnONPs allows the release of drugs within the target site over a prolonged period ranging from days to weeks [52], and the full resorption of the PRF membrane was not fully achieved after 14 days**,** thus the release of the trapped growth factors and the other osteoinductive stimulators will last for more than 14 days [53]. In this study, the superiority of the PRF/ZnONPs could be attributed to the ZnONPs synergistic effect with the PRF fibrin mesh in trapping and slowly releasing the growth factors and the other osteoinductive stimulators in the target site and subsequently accelerating bone growth and mineralization.

The significant differences in the size of the radiologic callus between the experimental groups could be attributed to the PRF clot itself. The intact PRF clot that fills and bulges from the CSD in the PRF group could contribute to the abundant callus formation. Meanwhile, in the PRF/ZnONPs group, the PRF membrane was perforated during the injection of ZnONPs and resulted in a reduction in the size of the PRF clot, and subsequently, moderate to minimal callus size was observed.

As mentioned by Mäkitaipale et al. [54], the growing rabbits have a high bone growth rate and their bone density is positively proportional with the age of the rabbit. So, for evaluation of the AOI density at each time-point, the measurement of the normal bone density was a must at each time-point where a significant increase in the density of the normal bone was observed and the AOI in the PRF/ZnONPs group had restored the normal density as early as the second month of the study.

The HU attenuation measurements applied in CBCT scanning can theoretically represent, describe and compare different bone densities in various sites [55, 56]. Regarding the selected CBCT acquisition parameters, Haiter-Neto et al. [57] reported that more accurate lesion depth estimates were obtained at 0.125-mm voxel size (but not 0.16, 0.25, and 0.36 mm). Depending on these results, a voxel size of 0.125 mm was used in this study.

The PRF group expressed the least AOI bone densities at all time-points while the PRF/ZnONPs group provided the highest measurements. To explain this phenomenon, we must know that the proliferation and differentiation of osteoprogenitor cells in secondary gap healing of CSD is proportional to the quality and the quantity of the collagen matrix where the larger the callus volume, the lower the bone density [58]. Results showed that the amount of the newly formed bone varied between the experimental groups; it was abundant in both PRF and PRF/ZnONPs groups versus little in the control group. So the control group showed higher densities than the PRF group. On the contrary, PRF/ZnONPs group showed the higher density despite the abundant collagen matrix and this could be attributed to ZnONPs which have osteogenic potential by stimulating collagen synthesis, alkaline phosphatase activity, synthesis of osteocalcin releasing zinc ions, and generating Zn-OH which acts as bone apatite nuclei and results in accelerated mineralization [12]. Additionally, Toledano et al. [36] mentioned that Zn Scaffold combinations provided the highest regenerative efficiency for bone healing by producing more branches and junctions at the trabecular bone and achieving a higher number of osteoblasts.

Normally, in spite of its apparently static structure, bone tissue is dynamic and undergoes a constant remodeling cycle controlled by a balanced coupling between bone resorption (by osteoclasts) and bone formation (by osteoblasts) to ensure a constant bone mass and to maintain skeletal integrity. It is of crucial importance that the amount of bone resorbed matches the amount of newly formed bone in each remodeling site [59]. Of note, experimental studies used mice Prx1 as a periosteal marker demonstrated that local periosteum and endosteum are the primary source of progenitor cells that differentiate directly into osteoblasts and contribute directly to fracture callus [60–62] and unfortunately, bone marrow-derived cells have a very minimal direct contribution to fracture healing [63]. Generally, after fracture, the release of inflammatory mediators; cytokines, growth factors, and prostaglandins, all of which lead to chemotaxis of these periosteal and endosteal osteoprogenitor cells toward the defect site [64]. Our findings demonstrated that the early BAD density of both PRF treatment groups was significantly less than the normal. This may be attributed to the high mediators and subsequently the high chemotaxis of the progenitor cells and osteoblasts toward the defect site instead of the normal bone. Additionally, the results of the control group confirmed this explanation where the least chemotaxis of the progenitor cells toward the defect allows the normal anabolic action in the bone adjacent to the defect. Moreover, the negative correlation between BAD density and the callus score confirmed this explanation. Generally, further studies using cell markers are needed to explain the certain cause of altered BAD density.

Most of the studies on rabbits’ radii and ulnae showed an evident fusion between the two bones and this was explained by Bodde et al. and Kasten et al. [65, 66] as a biologic natural response from cells of the surrounding tissues such as the periosteum remnants above and below the defect, in addition to the membrana interossea found between the two bones, might be responsible for the clear synostosis between the radius and the ulna in these bone regeneration researches. In this study, extensive fusion was observed in the cross-section of CBCT images between the ulna and adjacent radius which appeared as one bone and this was considered a major feature indicating incomplete remodeling of the defect healing. However, by the end of the study, a regression of the bone in the interosseous space and a reduction in the callus size in the PRF/ZnONPs group indicate better remodeling versus both PRF and control groups. The remodeling score was greatly dependent on the sum of the canalization, the bridging, and the callus scores. Results of this study showed that the signs of remodeling were appreciable in the animals of both PRF and PRF/ZnONPs groups but not in the animals of the control group. The passage of time was shown to have a significant impact on the remodeling between all experimental groups reflecting the anabolic and catabolic coupled bone repair process [67, 68].

## Conclusions

In conclusion, PRF enhanced reduction of the defect size, defect bridging by bicortical callus, recreation of marrow cavity, and remodeling while likely contributing to the formation of a huge callus of low density. The addition of ZnONPs to the PRF potentiates its effect by accelerating bone formation, and increasing bone quality and density.

### Clinical significance

This study illustrates the felicitous regenerative effects of both PRF and ZnONPs in critical segmental ulnar defects in rabbits that highlights the possible application as complementary therapies in different intraosseous wounds that do not heal spontaneously which may attribute to the osteoinductive, osteoconductive, and osseointegration properties of PRF and the mineralization potential of ZnONPs.

### Study limitations

Although PRF and ZnONPs exhibited great results in CSD bone regeneration, further histopathological, and histomorphometric examinations in addition to gene analysis are required to investigate their effects on CSD healing. Similar to other PRF studies, the application frequency should be verified with additional studies.

## Methods

### Animals and housing

A total of thirty healthy male New Zealand White rabbits, aged (mean ± SD) 4.0 ± 0.3 months and weighed 2.5 ± 0.5 Kg were allowed to acclimatize to their new conditions for 2 weeks before the start of the study and kept at the experimental animal room of the Surgery Department of Mansoura Veterinary Teaching Hospital, Mansoura University, Mansoura, Egypt, at a constant temperature of 22° ± 1 °C, 55% humidity, and a 12-h light/dark cycle. They had free access to a standard rabbit diet and water ad-libitum throughout the experimental period. Animals were managed according to the Guide for the Care and Use of Laboratory Animals approved by the Ethical Committee of the Faculty of Veterinary Medicine, Mansoura University, Egypt, and registration number (M/6) and all methods are reported in accordance with ARRIVE guidelines.

### Experimental design

In all rabbits (*n* = 30), a mid diaphyseal critical size defect was ostectomized in the right ulna. Then rabbits were randomly assigned into three experimental groups (*n* = 10): Control group where no materials were applied, PRF group, where the defect was filled with autologous PRF, and PRF/ZnONPs group where the defect was filled with ZnONPs based on PRF.

### Implants

#### Preparation of autologous PRF grafts

Autologous PRF was prepared from each rabbit in the PRF and PRF/ZnONPs groups by one step simple centrifugation (Hettich EBA 8S centrifuge, D-78532 Tuttlingen, Germany) of autologous 4 mL of whole blood in a sterile 5 ml vacuumed plain glass tube (Shanghai Goldenwell Medical Technology Co, Ltd., China) at a rate of 3000 rpm for 10 min (RCF = 402×g) at a 45° rotor angulation with a radius of 40 mm [69, 70]. The blood separated into three distinct layers; the upper acelluar plasma, the middle a strong leukocyte-rich PRF clot and the lower RBCs layer. The obtained PRF clot, was placed carefully into the ulnar bone defect with particular attention to PRF-bone contact.

#### Zinc oxide nanoparticles

ZnONPs dispersion with a concentration of 20 wt.% in H_2_O (Sigma-Aldrich chemicals no, Nasr City, Cairo, Egypt) was purchased and kept in a dark place at room temperature. The package contains 100 g of ZnONPs in a form of milky white color dispersion in a dark glass bottle (Table 3). ZnONPs dispersion was diluted to 0.2% according to Fielding et al. [38] using 0.9% normal saline.

**Table 3:** Specification of ZnONPs according to Sigma-Aldrich Co

**Characteristics**	**Structural color**	**Avg. particle size**	
ZnO	Milky white	≤40 nm (APS)	
**Concentration**	**Particle size**	**Density**	**pH**
20 wt.% in H_2_O	< 100 nm	1.7 ± 0.1 g/mL at 25 °C	7.5 ± 1.5

### Surgical procedures

The preoperative antibiotic regimen included intramuscular injection of Cefotaxime sodium (Cefotax, EIPICO pharmaceutical company, Egypt) at a dose of 50 mg/kg an hour before surgery. General anesthesia was achieved using an IM injection of 7.5 mg/kg xylazine (20 mg/ml; Xylaject; Adwia Co, Egypt) and 35 mg/kg ketamine hydrochloride (Ketamine 50 mg /10 ml, Rotexmedica, Germany).

**Fig. 7:**
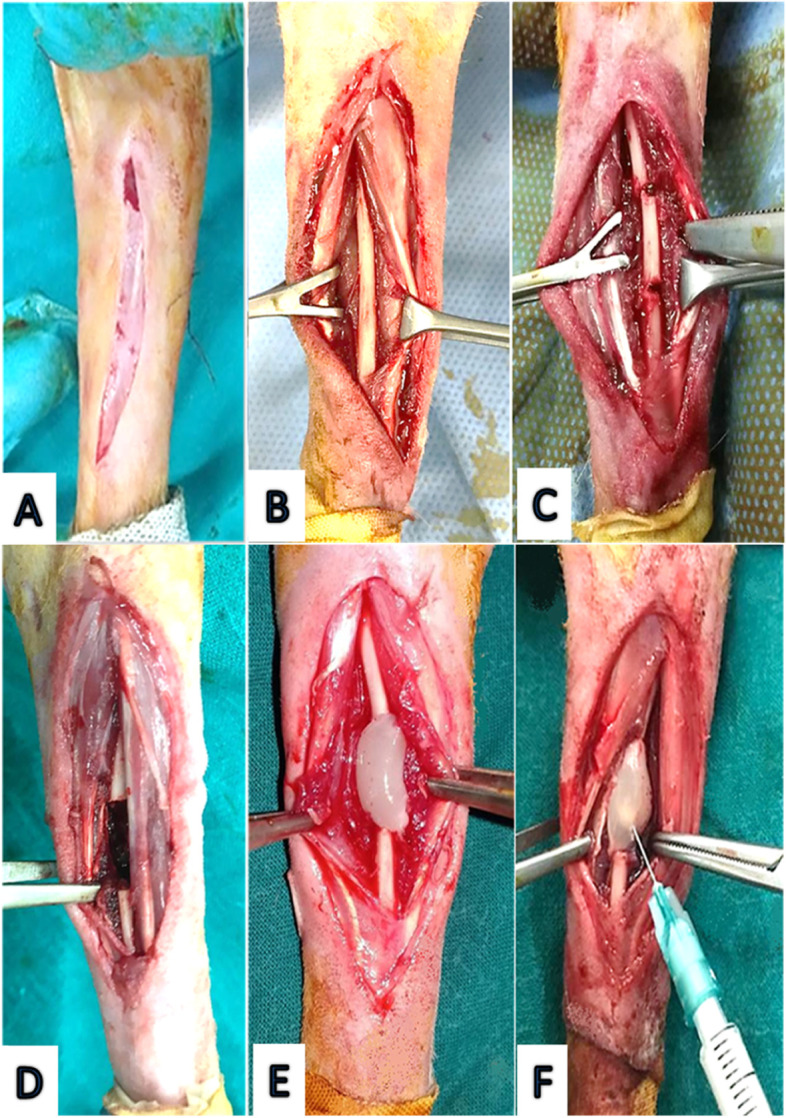
The surgical procedures for induction of 12 mm critical size ulnar defect. A 5 cm long linear incision was made over the craniolateral aspect of the mid of the antebrachium (**A**). The ulna was exposed by retraction of the surrounding muscles (**B**) where 2 drilling holes with 12 mm apart were created in the mid-diaphysis (**C**). The defect was left empty in the control group (**D**), filled with PRF clot in the PRF group (**E**) or ZnONPs were inoculated into the PRF clot in the PRF/ZnONPs (**F**)

On lateral recumbency and under complete aseptic preparation, a 4–5 cm long incision was made over the craniolateral aspect of the mid of the antebrachium where both subcutaneous tissue and antebrachial fascia were opened carefully. Using a periosteal elevator, the ulna was separated from the attached muscles then the bone was exposed by retraction of the muscles. Using a low-speed electric drill (APT, China) with a 1.5 ml diameter drill bit, A 12-mm segmental diaphyseal defect was ostectomized from the mid-shaft of the ulna under irrigation with 0.9% sterile saline solution. The periosteum was removed with the bone and great care was taken to avoid muscle damage. The defect was flushed using sterile normal saline solution (Fig. 7).

In the control group, the defect was left empty while in both PRF and PRF/ZnONPs groups, PRF clot was used to fill up the defects and particular attention was paid to ensure graft bone contact. In the PRF/ZnONPs group, 0.1 ml of ZnONPs dispersion 0.2% (2 mg ZnONPs)**,** was inoculated carefully into the PRF clot using an insulin syringe. Closure of the antebrachial fascia over the defect preceded closure of the subcutis using 3–0 vicryl (ETHICON, USA) then the skin was sutured using 3–0 silk (ETHICON, USA). A protective bandage was applied.

### Postoperative management and follow-up

All rabbits were allowed to recover in an area warmed to 37 °C until they were fully awake. Afterward, they were moved to their cage. All animals received meloxicam (Anticox II 15 mg, ADWIA, Egypt) in a dose of 0.6 mg/kg for 5 days. The preoperative antibiotic regimen continued for 5 days. The incisions were dressed daily with povidone-iodine and bivatracin antibiotic local spray (ECAP, Egypt) then re-application of protective bandages was done. The feeding condition, weight, body temperature, and breathing were investigated daily. Additionally, animals were observed daily to evaluate the status of cutaneous wound healing and the movement function of rabbits till the end of the study.

### Radiologic evaluation of healing

Both quantitative and qualitative bone healing evaluation criteria have been utilized to build a picture for the progression of healing up to 90 days in vivo PO period. Cone-beam computed tomography (CBCT) was performed on the 30th, 60th, and 90th PO days using an orthodontic-grade CBCT scan (120 kV, 5 mA; i-CAT FLX V17–19; Imaging Sciences International, Hatfield, USA), at a clinically typical 0.125-mm voxel size and FOV dimensions of 16 cm diameter х 4 cm height. 3D images were reconstructed from the series of 2D projections using the classical filtered back-projection algorithm. The On-Demand 3D software app version.1.0.7510 (Cybermed, Korea) was used to produce 3D volume renderings. Visualization options included multiplanar orthogonal (coronal, axial, and sagittal) viewing angles. The Area of interest (AOI, the rarified bone in-growth) was identified visually from the original dense structure of the ulnar cortical plates based on observing longitudinal serial slices throughout the sample.

Compared to the baseline defect size (12 mm), all the CT images were visually analyzed by two independent reviewers in each group at each time-point for the presence of new bone formation and reduction of the defect size, bridging of the gap, callus size, and canalization of the marrow cavity. These criteria (Table 4) were determined according to modified scoring systems [65]. The remodeling score was calculated as the sum of the canalization, bridging, and callus scores.

**Table 4:** Modified radiological scoring system (Bodde et al. [65])

Score		Description
**Reduction in defect size**	0	No healing
	1	Less than 25% reduction
	2	25–50% reduction
	3	50–75% reduction
	4	More than 75% reduction
	5	No gap
**Canalization score**	0	0–25%
	1	25–50%
	2	50–75%
	3	75–100%
**Bridging score**	0	No bridging
	1	Partial/unicortical bridging
	2	Complete/bicortical bridging
**Callus score**	0	Extensive callus
	1	Moderate callus
	2	little/minimal callus
	3	No callus
**Remodeling score**	**Sum of these scores**	

The bone mineral density of the original dense structure of the normal ulnar cortex, regenerated bone in-growth (AOI), and the bone adjacent to the defect (BAD) were quantified using a quantitative CT method by Houn’s Field units based on Itoi et al. [21]. Due to the non-uniformity of the bone mineral density in the same CT image, five-point densities were measured with a specified 5 × 5 mm^2^ diameter region of interest (ROI) for each point and the mean ± SD was calculated. The mean of AOI densities of each group at each time (30th, 60th, and 90th PO days) was statistically compared with the total mean of the normal densities of the ulnar bone at the same time.

### Statistical analysis

All statistical analyses were carried out using SPSS software program version 25. Both quantitative values (bone densities) and semi-quantitative values (percent of defect filling) were expressed as mean ± standard deviation (SD) and were assessed using normal probability plots. Non-parametric qualitative data (reduction in the defect size, bridging score, canalization score, callus score, and remodeling score) were expressed as median (minimum-maximum) and were assessed using Kruskal–Wallis nonparametric ANOVA. To assess the effect of PRF and ZnONPs, a one-way analysis of variance (ANOVA) was used to analyze the data followed by Tukey-Kramer HSD for multiple comparisons. Results were considered significant when *P* ≤ 0.05. In addition, Spearman’s correlation coefficient was computed to assess the relationship between the BAD density and the extent of the callus in the CBCT.

## Data Availability

Not available.

## Abbreviations

ZnONPs: Zinc oxide nanoparticles
PRF: Platelet-rich fibrin
PO: Postoperative
CSD: Critical size defect
CBCT: Cone beam computed tomography
AOI: Area of interest
HU: Hounsfield units
BAD: Bone adjacent to the defect
ROI: Region of interest
